# Screening of Toxic Effects of Neonicotinoid Insecticides with a Focus on Acetamiprid: A Review

**DOI:** 10.3390/toxics11070598

**Published:** 2023-07-08

**Authors:** Lucia Zuščíková, Denis Bažány, Hana Greifová, Nikola Knížatová, Anton Kováčik, Norbert Lukáč, Tomáš Jambor

**Affiliations:** Institute of Applied Biology, Faculty of Biotechnology and Food Sciences, Slovak University of Agriculture in Nitra, Tr. Andreja Hlinku 2, 949 76 Nitra, Slovakia; xbazany@uniag.sk (D.B.); hana.greifova@uniag.sk (H.G.); nikola.knizatova@uniag.sk (N.K.); anton.kovacik@uniag.sk (A.K.); norbert.lukac@uniag.sk (N.L.); tomas.jambor@uniag.sk (T.J.)

**Keywords:** neonicotinoids, acetamiprid, toxicity, reproduction

## Abstract

Recently, neonicotinoids have become the fastest-growing class of insecticides in conventional crop protection, with extensive usage against a wide range of sucking and chewing pests. Neonicotinoids are widely used due to their high toxicity to invertebrates, simplicity, flexibility with which they may be applied, and lengthy persistence, and their systemic nature ensures that they spread to all sections of the target crop. However, these properties raise the risk of environmental contaminations and potential toxicity to non-target organisms. Acetamiprid is a new generation insecticide, which is a safer alternative for controlling insect pests because of its low toxicity to honeybees. Acetamiprid is intended to target nicotinic acetylcholine receptors in insects, but its widespread usage has resulted in negative impacts on non-target animals such as mammals. This review summarizes in vivo and in vitro animal studies that investigated the toxicity of specific neonicotinoids. With summarized data, it can be presumed that certain concentrations of neonicotinoids in the reproductive system cause oxidative stress in the testis; spermatogenesis disruption; spermatozoa degradation; interruptions to endocrine function and Sertoli and Leydig cell function. In the female reproductive system, acetamiprid evokes pathomorphological alterations in follicles, along with metabolic changes in the ovaries.

## 1. Introduction

Since the beginning of the industrial revolution and urbanization, the global environment has been continually changing, and, today, it has become a major source of different hazardous substances. Numerous xenobiotic substances, including residues from personal care products, agrochemicals, triazines, chlorinated compounds, polychlorinated biphenyls, polycyclic aromatic hydrocarbons (PAHs), and phosphor organic pesticides, adversely affect our environment because of their long-term persistence and slow degradation in the ecosystem [1].

Several reports state a massive presence of harmful xenobiotics compounds in the air, soil, and water [2,3,4]. The use of agrochemicals in agricultural practice results in the release of contaminants into groundwater, which contaminates irrigation sources. In addition, the Federation of Indian Chambers of Commerce & Industry [5] demonstrated that the routine use of agricultural chemicals may impact over 90% of non-target species, and some peri-urban areas have particularly high rates of water pollution. The persistence of agrochemicals in soil is closely related to their contamination of groundwater. Whether a pesticide will leach into groundwater depends on its ability to absorb. The agrochemicals with poor adsorption or absorption on the surface of soil will seep into the groundwater and contaminate it [6]. At the same time, the excessive accumulation in the soil is stimulated by plenty of anthropogenic activities, including fuel combustion, fertilizer application, and pesticide usage. Subsequently, agrochemicals are able to persist in the soil profile for a long period, whereas the rate of decomposition depends on climate conditions, soil type, and physicochemical properties [7,8]. In an attempt to solve the problem of weeds and pests, the indiscriminate use of agrochemicals is gradually affecting soil diversity, mineral composition, the maintenance of soil structural properties, and overall health. The concerns about the potential negative impacts on human and wildlife health are justified, and the questions about the tightening of current legislation are appropriate [9]. As we have already indicated, the widespread adoption of mechanization, irrigation techniques, and novel agricultural practices a couple of years ago resulted in increasing gains in the production of agricultural crops. At the same time, it is obvious that the dramatic growth in cropland productivity has been supported by the massive use of agrochemicals, which causes concern among the majority of the population. However, manufacturers of these compounds and other chemical industry advocates declare that agrochemical applications are essential for producing enough food, particularly given the growing global population [4,10].

In general, agrochemicals are compounds used to control weeds and diseases in crops during many agronomic practices, and they have become a key tool in crop protection. If we take into account that global crop losses are caused by 50,000 plant pathogen species, more than 9000 mite species, and approximately 8000 weed species, reasonable applications of agrochemicals are definitely important. According to the last FAO report published in 2022, the total amount of agrochemicals used in agriculture remained relatively stable in 2020. It is estimated that more than 2.68 million metric tonnes of active substances have been applied. At the same time, the worldwide application of agrochemicals per area of cropland was estimated as 1.8 kg/ha and 0.37 kg/person [11]. The global application of individual types of agrochemicals varied across the last decade. A moderate growth in the use of herbicides has been confirmed, while fungicides and insecticides were applied sparingly. Agrochemicals include herbicides, fungicides, rodenticides, nematicides, and insecticides, which are frequently used due to the growth in the occurrence of pests in the agricultural sector [12]. This was also confirmed by a recent study. Based on the reported statistics, pathogen-related losses ranged from 10% to 28% in wheat, from 20% to 40% in maize, and from 11% to 32% in soybeans globally. This is the reason why the intensity of crop protection increased 15–20-fold in agrochemical usage around the world [13]. According to the previous report from the Food and Agriculture Organization [14], the most currently used agrochemicals are herbicides (56%) and fungicides (25%), followed by insecticides at approximately 20%. Insecticides are classed chemically as organochlorine, carbamates, pyrethroids, and neonicotinoids (NEOs) and include other classes such as spinosyns or benzolureas. From the perspective of common applications, organophosphates, followed by NEOs, are the most often used. Neonicotinoids, as a relatively new class of insecticides, are widely used in both urban and agricultural settings [15,16]. Exposure to them has been linked to a couple of short-term and chronic health effects, such as neurological development issues, liver pathologies, and genotoxic and carcinogenic effects, as well as a potential to induce adverse impacts on reproductive development [16].

Currently, the possible effects of these chemicals on the physiological processes of individual organisms are still not fully understood. In addition, knowledge about the impact of exposure to NEOs, especially acetamiprid, on the reproductive system is limited as well as inconsistent and does not provide a relevant background for solving this problem. Due to this, the purpose of this critical review is to gather new information about the risks of NEOs and to help highlight the toxicity, mechanism of action, and other harmful aspects of acetamiprid’s presence in our environment. It is necessary to look at the area of impact of these substances on the reproductive system, which is very sensitive to NEOs’ effects. More than 200 publications were reviewed, of which more than 100 are cited here, to provide a comprehensive overview of their interaction with human and animal health.

In recent years, there has been increasing concerns over extensive environmental threats of NEO insecticides [17]. These substances were discovered in the 1980s, and in the early 1990s, the first commercial compound, imidacloprid, was made available. After that, NEOs rapidly spread due to their properties like their systemic mode of action and high effectiveness at low dosages and because of their supposedly low toxicity to vertebrates, including humans [18]. In comparison to other pesticides, NEOs are easily absorbed by plants and react swiftly at low concentrations. Some of these active compounds are approved for seed treatments (clothianidin), for foliar sprays (acetamiprid and thiacloprid), and for both applications (imidacloprid and thiamethoxan) [19]. The extensive application of NEOs includes pest control and vector control for veterinary and domestic uses [20]. These insecticides are strongly efficient at controlling sap-feeding insects and insects feeding on plant tissues [21].

**Figure 1:**
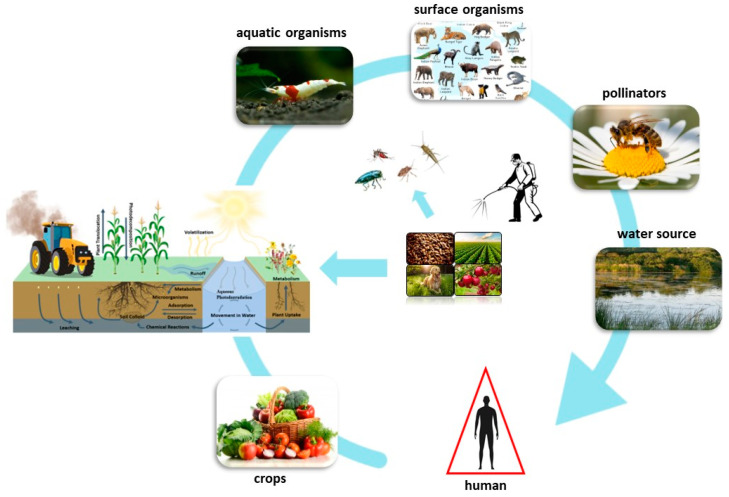
The environmental fate and routes of neonicotinoid pesticide exposure after application.

This type of insecticides was created to have a similar structure to nicotine’s, and they both affect the cholinergic neurotransmitter system. It was previously believed that NEOs had a limited biological half-life. However, recent research has revealed that they can persist in the environment for up to 19 years [22,23]. They are water soluble at pH 7 and 20 °C at quantities of 184–590.0 mg/L. Consequently, NEOs are absorbed and transported all over the plant´s system to provide protection against insects [24]. Only around 10% of the applied insecticide reaches the target organisms; the remaining 90% is dispersed into the environment, where it may have negative effects on ecosystems and non-target organisms (Figure 1) [25].

Despite their harmful effects to honeybees, NEOs have been presumed to be safe replacements for organophosphate pesticides, carbamates, and pyrethroids [26]. It was proved that they have been frequently found in substances like water, soil, and dust; animal products; and organisms like insects, birds, and even humans [27]. According to the literature, NEOs have acute or subacute toxic effects [28,29] on non-target organisms, which are related to exposure route and time [30,31]. Because NEOs are widely utilized in the environment, they provide significant health concerns to humans and ecosystems, affecting the ecological balance [32], disturbing female reproduction [33], and disturbing the development of human nervous systems [16]. Even though NEOs are less hazardous to mammals and people than traditional insecticides, negative effects on animals and human health have been confirmed.

Although pesticides like NEOs are essential for the steady supply of agricultural products, it is important to closely monitor their effects on human health by assessing food; their persistence in the environment, including agricultural soils and groundwater; and their ecological effects on beneficial insects like bees. People are now more interested in the numerous risks associated with consuming these substances because of reports on their negative effects. Naturally, monitoring pesticides like NEOs, which can persist in a wide range of matrices, requires very sensitive, highly selective, and accurate analytical methods. Sample preparation, separation, and quantification are the three steps that form a general analytical procedure for pesticide residues. Target pesticides must be “extracted” from a sample in the previous sample preparation phase, and interfering elements must be removed from a sample extract, a process often known as “clean up”. In the latter, gas chromatography (GC) or high-performance liquid chromatography (HPLC) are used to separate the target insecticides. Following that, they are both qualitatively and quantitatively assessed using detectors of various sorts that correlate to the properties of the target pesticides. Both qualitative and quantitative data can be obtained simultaneously using a mass spectrometer (MS) or tandem MS (MS/MS) as the detector [34].

### 1.1. Classification of Neonicotinoids

**Figure 2:**
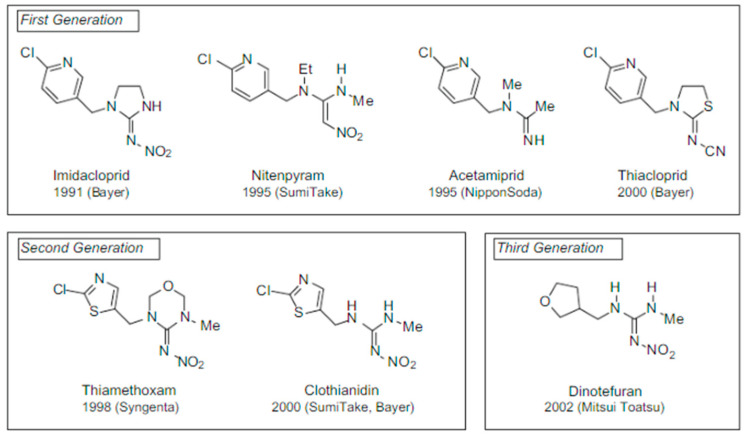
Chemical structures of common neonicotinoids (Content may be subject to copyright) [42].

Chemically, NEOs have a comparable structure to nicotine, which has natural insecticidal properties [35]. Like any pesticide class, NEOs can be divided into a number of distinct chemical subclasses depending on their molecular structures (Figure 2). They are classified as hydroheterocyclic guanidines/amidines with functional substituents. The chemical structure of NEOs consists of four common elements: an aromatic heterocyclic group, elastic bonds, hydroheterocyclic or guanidine/amidine groups, and an electron withdrawing group. Additionally, new NEO derivatives are continually being developed by altering the structures of the mentioned compounds by substituting a sulphonamide functional group or its cyclical similar version for a cyano- or nitroguanidine/amidine group [36]. These new derivates work as agonists on postsynaptic insect nicotinic acetylcholine receptors (nAChRs) [37]. Seven NEOs are commercially available including imidacloprid, acetamiprid, thiacloprid, thiamethoxam, clothianidin, dinotefuran, and nitenpyram. There are three generations of NEOs, including chloropyridinyl compounds (the first generation: imidacloprid, nitenpyram, acetamiprid, and thiacloprid), chlorothiazolyl compounds (the second generation: thiamethoxam and clothianidin), and tetrahydrofuryl compounds (the third generation: dinotefuran), and other newly developed NEOs [38]. Also, subclasses can be simply divided into two types: nitroguanidine and cyanomidine. Nitroguanidine NEOs have N-nitro groups in their structure with oxygen atoms, which makes them more polar and reactive (imidacloprid, thiamethoxam, and clothianidin). On the other hand, cyanoamidine NEOs contain cyanoamidine groups in their particles, which have less polar and reactive properties than nitro groups (acetamiprid and thiacloprid) [39,40]. The first generation of NEOs behave like a partial agonist of the nicotinic nAChRs. The second generation of NEOs acts in different ways from the first generation. They are poor agonists of insect nAChRs. Although it causes a significant depolarization at cercal afferent/giant interneuron synapses, it is a complete agonist of them. The third generation of NEOs can interact with nAChRs in insects. For example, dinotefuran (third generation) can have nerve-excitatory activity that is similar to clothianidin (second generation) but less potent than imidacloprid (first generation). In the case of nerve-blocking activity, this chemical is more similar to imidacloprid and modestly higher than that of clothianidin [41].

### 1.2. Mode of Action

The main receptor for insect nervous systems is nicotinic acetylcholine receptor (nAChR), which is a target for NEOs, nicotinoids, and other insecticides [43,44]. nAChR is a ligand-gated ion channel in the central nervous system of invertebrates that mediates excitatory cholinergic neurotransmission and is the target location for NEOs. NEOs, spinosyns, and nereistoxin analogues are the three main types of insecticides that act by interfering with the normal physiological functioning of the nAChR [45]. The nAChR consists of hetero- or homopentamer subunits arranged centrally around a cation-selective pore. Each nAChR heteropentamer has two orthosteric binding sites, which occur in the extracellular domain at the interface of α adjacent and β subunits, donating loops A to C and loops D to E, respectively, for the substrate interaction [46]. When two α or three non-α subunits make up nAChRs, the α subunit provide loops A, B, and C, while the non-α subunits provide loops D, E, and F to create the orthosteric site [47]. These receptors are placed all over the central nervous system of insects. The biphasic response elicited by NEOs increases the frequency of spontaneous discharge and blocks nerve propagation [48].

**Figure 3:**
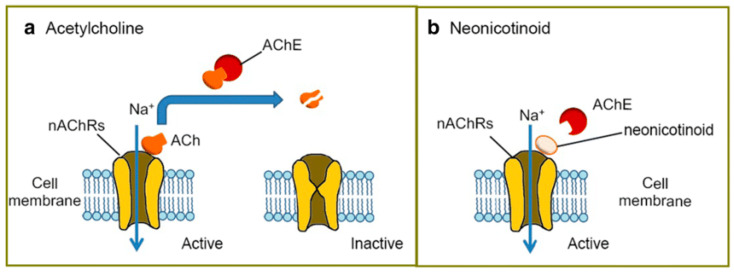
The action of nicotinic acetylcholine receptors in the presence of acetylcholine (**a**) and a neonicotinoid (**b**) [62].

Additionally, there are many nicotinic receptor subtypes found in mammalian tissue. These receptor subtypes are created from various combinations of nine α, four β, γ, δ, and ε subunits [49]. In mammals, nicotinic receptors are found in the autonomic ganglia, skeletal muscles, spinal cords, and several brain areas [50,51]. Insecticidal action is increased by including synergists that block oxidative degradation [48]. These insecticides bind to nAChRs in the postsynaptic neuron, where they act as a “false transmitter” (agonist) (Figure 3). While nicotinoids are structurally similar to NEOs, they are differentiated by the presence of an ionizable basic amine or imine substituent [44]. Acetylcholine (Ach) causes the opening of channels for Na^+^ influx and K^+^ efflux [52,53]. The replacement of Ach at the agonist site results in NEO toxicity. This interference with Ach neurotransmitter communication causes the activation of the receptor, resulting in neurotoxicity symptoms/signs. The activation of nAChRs typically leads to an increased release of important neurotransmitters such as dopamine, serotonin, glutamate, and GABA (γ-aminobutyric acid) [54]. nAChRs are present in the peripheral and central nervous systems of humans. They send messages to the neurological system that cause skeletal muscles to contract [55,56]. Fully functional nAChRs are also found in bronchial epithelial cells, endothelial cells, lymphocytes, keratinocytes, cochlear hair cells, and chromaffin cells [57]. In electronegative sites, NEOs bind to nAChRs, which adds to their toxicity for insects [36,58].

It has been discovered that NEOs are typically found in foods. An in vitro study showed that imidacloprid and other NEOs can directly activate and alter the nAChR α4β2 subtype in humans. This is a well-known subtype of nAChR in mammal brains and contains the highest density of receptors in the diencephalon. Many aspects of brain activity, including cognition, memory, and behaviour, are associated with the nAChR subtype α4β2 [59]. Typically, ligand binding interactions between the receptors and standard agonists/antagonists are used to evaluate the binding characteristics and densities of insect nAChRs [60]. NEOs are considered to be partial/full agonists of insect nAChRs and weak agonists of mammalian receptors. At saturation doses, full agonists activate the channels to about 100% open probability (for example, acetylcholine). Partial agonists will trigger less of a total current than full agonists at the same binding site. Also, they can prevent endogenous or exogenous full agonists from activating receptors [61].

Several proteins are functionally related to nAChR. One of these proteins is peripheral membrane protein (43-kDa) rapsyn, which is involved in receptor–cytoskeleton connections. Rapsyn acts on receptors and binds with all subunits, making it essential for nAChR clustering in muscle. Rapsyn was additionally found in non-muscle cells such as ciliary ganglia neurons, cardiac cells, fibroblasts, and Leydig cells [57].

### 1.3. Toxicity to Non-Target Organisms

Because of their widespread usage, NEOs are occurring more frequently in terrestrial and aquatic environments, resulting in their leaching into water and the accumulation of residues in soil. Due to NEOs being widely used in the environment, they present considerable danger to non-target fish, birds, mammals, and even humans (Table 1) [63]. The toxicological impact of NEOs was observed in fish and other aquatic microorganisms, in which they induced genotoxicity and reductions in immune system function. These substances can affect land animals via direct contact or food chain transfer. Molluscs and earthworms are especially vulnerable to pesticides because they come into direct contact with a large area of soil with their bodies. Earthworms´ oxidation and degradation of lipids, proteins, and nucleic acids have been reported in response to high doses of dinotefuran [64,65]. The type of soil where NEOs are applied has a significant impact on environmental fate and their dissipation. Under the proper conditions, NEOs can persist in soils. The dissipation time is influenced by a variety of factors, including low levels of soil quality, microbial activity, temperature, and precipitation. In one recent study, the soil mobility of two specific NEOs was observed. The results provide further evidence of the mobility of NEOs and show that NEO concentrations in leachates are affected by a variety of factors, such as the type of soil, in addition to the compound type [66].

#### 1.3.1. Neurotoxicity

Primarily, NEOs work as nAChR agonists, which are responsible for the majority of excitatory neurotransmissions in an organism’s nervous system. Since the nervous system is NEOs’ main target of action, early investigations focused on how they affected the neurological systems of individuals. Several reports observed NEOs’ effects on various animal models (Table 2.).

#### 1.3.2. Organotoxicity

The liver is the primary target of NEOs since its main function is metabolism and toxin removal. Nevertheless, only high dosages of NEOs generally produce evident liver damage, which is accompanied by decreased food intake and weight loss [16]. Bhardwaj et al. [73] demonstrated the toxic effect of imidacloprid on female rats fed orally. At higher concentrations, the kidney, liver, and brain reveal pathological impacts. Moreover, some parameters significantly increased, glutamate pyruvate transaminase and glutamate oxalacetate transaminase activity and glucose and blood urea nitrogen content. A similar hepatotoxic effect was observed in a study conducted by El Okle et al. [74], in which they administered thiamethoxam to rabbits. Hepatotoxicity was reflected in increased levels of bilirubin and alterations in liver tissue, including the necrosis and apoptosis of hepatocytes, lymphocyte infiltration, and fibrosis induced by cell death.

#### 1.3.3. Other Toxicity Impacts

Other toxicity impacts are summarized in Table 3.

## 2. Characterization of Acetamiprid

The first commercial product containing acetamiprid was registered in 2002 for crops and cattle, even though acetamiprid was first synthesized in 1984 [81]. Acetamiprid (ACE (N-((6-chloropyridin-3-yl) methyl)-N′-cyano-N-methylethanimidamide)) is an NEO pesticide used to regulate insects on leaves, fruits, vegetables, and ornamental plants [82,83]. Nippon Soda started producing ACE in Japan in 1995 [55]. In Europe, the United Kingdom, and the United States of America, ACE is sold as water-soluble granules under the trade names Mospilan, Autentic, Gazelle (containing 20% ACE), and Assail (containing 30% ACE) (ACE is also available under a number of other trade names on the global market). According to data that were available up until 2009 (in some cases, until 2012), ACE had a small market share compared to other NEOs (about 10.5% of global sales) [41,84]. On the other hand, there is very little current data on the amount of ACE that is now utilized and sold globally. Additionally, due to the implementation of European laws, its proportion is expected to increase over the next few years in many European countries [85]. While ACE can enter the terrestrial environment by rain, irrigation, or other routes, it is not directly applied to soil. Since ACE is highly soluble in water, it has the potential to contaminate food supplies in the environment [86,87]. The main source of exposure is via food and water. Another exposure route is via skin contact or inhalation for individuals working with ACE.At atmospheric conditions, ACE is resistant to hydrolysis and photodegrades slowly in water [88]. Residues can accumulate in soil, with a half-life of 23 days in the field and 450 days in laboratory settings, impacting soil organisms [89]. In the central nervous system of insects are postsynaptic nicotinic acetylcholine receptors, where ACE can bind [90]. Exposure to ACE causes change in membrane potential, disrupts nerve transmission, and ultimately leads to neuronal hyperexcitation, paralysis, and finally death in insects. Even though acetamiprid is extremely toxic to insects, it has been proven that this substance also has a high affinity for mammalian nicotinic acetylcholine receptors. According to certain findings, exposure to acetamiprid inhibits the mRNA expression of the α3, α4, and α7 nicotinic acetylcholine receptors in cerebellar cells in rats and brain regions and testis in mice. Nicotine acetylcholine receptors are mostly located in the neuromuscular and reproductive systems of mammals [91].

Direct and indirect exposure are highly correlated with the accumulation of ACE in numerous tissues [92]. Residues of acetamiprid have been reported in the urine [93,94], brain, digestive tract [95], liver, and testis of mice [96]. After oral ingestion, ACE is delivered to many body organs via the bloodstream, passing through the blood–brain and blood–testis barriers [97]. Ingested ACE is metabolized by the liver and ultimately excreted by the kidney. As a result of exposure to environmental toxins, the liver, a target organ for detoxification, is vulnerable to a number of illnesses. NEOs like ACE are quickly metabolized in the liver by phase enzymes such as aldehyde oxidase (AO) and cytochrome P450 (CYP) in mammals [98].

As previously mentioned, the majority of NEOs, including ACE, are eliminated in the urine. Because of NEOs’ water solubility and low molecular weight, they appear to easily pass through the glomerulus. Thus, the stress of these injury chemicals in the body causes damage to both the liver and kidneys [99]. The absorption of ACE in live tissues has not been studied in detail. In one study, the effects of temperature and ACE concentration in human colon carcinoma cells were investigated. Lower temperatures reduced ACE absorption via apical-to-basal and basal-to-apical routes. Remarkably, the basal-to-apical pathway was concentration-independent, but the apical-to-basal pathway was concentration-dependent. This finding indicates that certain membranous transporters are involved in ACE absorption inside the cells [100]. In living tissue, ACE is known to be easily decomposed by the demethylation, deacetylation, and hydrolysis of the cyano–imine bond. Ueyama et al. [101] analysed the urine samples of 50 children (<3 years old) with no direct exposure to ACE. In total, 39 samples were presented ACE metabolite (N-desmethyl-acetamiprid) that implied exposure via environmental pollution. The N-desmethyl-acetamiprid metabolite was shown to be the most prevalent and easily detectable metabolite in other investigations that also revealed ACE in numerous tissues and fluids [95].

### 2.1. Acetamiprid Toxicity

Due to its widespread and ongoing usage, ACE has been identified as an environmental toxicant. Also, exposure to ACE causes organ system toxicity, disrupting immune physiology, ion balance, and behaviour. Easy solubility and fast biological interaction make live tissues more vulnerable to ACE exposure. The LD50 (LD—lethal dose) for oral exposure is identified to be 200–220 mg/kg b.w. (body weight) in rats [102,103,104], while LD50 for exposure by drinking water is identified to be 1000 mg/kg b.w in rats [105,106]. Several animal studies have demonstrated dose-dependent ACE toxicity and suggested the production of oxidative stress in various organs. Subchronic oral ACE exposure was observed to cause oxidative-stress-mediated structural changes in the liver and kidney, as well as haematological and biochemical changes in rat hepatic tissue in a dose-dependent manner [107,108]. Exposure to ACE has also been linked to ion imbalances inside the cells when, specifically, ACE raised cerebellar Ca^2+^ levels, implying the inhibition of Na^+^/K^+^, Ca^2+^ and Mg^2+^ ATPase activity in rat brain [109]. It has been reported that ACE reduced antioxidant enzyme activity and generated inflammation, necrosis, and apoptosis in the liver and kidneys of rats.

#### 2.1.1. Oxidative Stress

In vertebrates and invertebrates, NEO-induced damage to lipids, DNA, and proteins may be caused by oxidative stress, reactive oxygen species (ROS), and reactive nitrogen species (RNS) [110]. It has been demonstrated via numerous in vitro (Table 4) and in vivo studies that exposure to ACE causes considerable ROS production, which unbalances both enzymatic and non-enzymatic antioxidants.

As free radicals interact with cells, they start a series of events that cause damage by oxidizing structural components and depleting the antioxidant capacity. These free radicals contribute to the development of oxidative stress and many pathogenic processes. Previous research showed increases in malondialdehyde and liver enzyme levels, decreases in glutathione content, and histopathological changes in the liver and kidneys of rats orally fed with ACE [108]. In addition, a recent study found that mice exposed to ACE had significantly higher levels of malondialdehyde, phenylalanine, and branched chain amino acids in their liver tissue, as well as lower antioxidant activity. This suggests that lipid accumulation may be the cause of the generation of oxidative stress due to disturbances in amino acid metabolism [114].

#### 2.1.2. Apoptosis and Genotoxicity

In addition to causing oxidative damage, ACE has been shown to act as a mutagen, alter gene expression, and interfere with cell apoptosis. Apoptosis is a process that results in the controlled death of cells, regulated by pro- and anti-apoptotic proteins. The main inducers of intrinsic apoptosis include oxidative stress, DNA damage, and altered gene expression. It has been proven that ACE exposure causes in vitro genotoxicity, cytotoxicity, and consequent DNA damage as well as molecular damage. Apoptosis has several negative effects on the genetic material and cellular balance of the tissue. In one study, Annabi et al. [111] discovered a significantly negative impact on cell viability, increasing DNA damage and apoptosis (caspase-dependent) in PC12 cells treated with ACE (100–700 M). In another study, Senyildiz et al. [115] observed DNA damage in human neuroblastoma cells (SHSY-5Y) and human hepatocellular carcinoma cells (HepG2) after ACE treatment in a dose-dependent manner.

#### 2.1.3. Reproductive Toxicity

Reproduction is one of the most sensitive processes of organisms to environmental and occupational contaminants, particularly pesticides. It has been reported that insecticides cause oxidative stress in the testis; spermatozoa degradation; spermatogenesis disruption; interruptions to endocrine function and Sertoli and Leydig cell function at any level of hormonal control [116].

Primarily, ACE has a neurotoxic mechanism of action, and early studies focused mostly on its acute toxicity to the mammalian nervous system. It can also, however, have negative impacts on other body systems. ACE has been confirmed to be damaging to reproduction in several species. A previous study investigated the toxic effects of ACE on reproduction [117]. The samples of ACE were orally given to male rats in concentrations of 12.5 mg/kg, 25 mg/kg, and 35 mg/kg for 90 days. The results showed that plasma testosterone levels and sperm count both decreased in a dose-dependent manner. Additionally, it was proven that ACE inhibited the synthesis of adenosine triphosphate (ATP) and cyclic adenosine monophosphate (cAMP) in Leydig cells [118]. The application of ACE may cause increasing concentrations of reactive oxygen species (ROS) in the testes, decreasing the overall weight of testosterone-responsive organs, the concentration of serum testosterone, and sperm quality [96]. Moreover, it has been proven that ACE induced reproductive toxicity in male guinea pigs. The administration of ACE induced a reduction in the weight of the testes, epididymides, vas deferens, and supporting glands. The testicular structure, testosterone concentration, testosterone response time, and epididymal sperm characteristics were also negatively affected [119].

Sub chronic exposure to ACE can cause reproductive destruction, decreasing in the motility, count, and viability of sperm as well as reductions in serum testosterone and gonadotropin-releasing hormone levels in male rats [120]. A previous experiment on mature rats observed how ACE administered by oral gavage affected them. The results showed decreases in the numbers of spermatids, epididymal sperm, and Leydig cells, together with decreases in the expression levels of Star (steroidogenic acute regulatory protein), Cyp11a1, Hsd3b (3-β-hydroxysteroid dehydrogenase), and testi weight. Also, the morphology of seminiferous tubules was changed in all rats in a dose-dependent manner [103]. It has been reported that NEOs (especially ACE) affect female reproductive system health by inducing pathologic morphological abnormalities in ovarian follicles, reductions in embryo quality, and metabolic changes in ovaries [121]. Additionally, the administration of ACE to pregnant rats during the organogenesis phase caused a developmental toxic impact in the way of morphological, soft tissue, and skeletal abnormalities [122]. Moreover, Babeľová et al. [123] discovered that when prokaryotic-stage mouse embryos were treated with NEOs (acetamiprid, thiacloprid, thiamethoxam, and clothianidin) and associated product solutions, all NEO insecticides at 100 μmol/L had a negative impact on mouse embryo development. At a concentration of 10 μmol/L, ACE and thiamethoxam both lowered blastocyst quality.

## 3. Conclusions

Neonicotinoids are a class of chemicals that are widely utilized as insecticides and veterinary medicines around the world. Investigating neonicotinoids’ harmful effects and toxicological mechanisms is necessary to protect non-target species and people from harm. As an example, the insecticide acetamiprid, a new-generation chloronicotinyl, is frequently used as a less toxic alternative to carbamates and organophosphates to manage insect pests. Although acetamiprid is intended to target the nicotinic acetylcholine receptors in insects, its widespread use has had negative consequences on mammals and other non-target organisms. It is persistent in the environment, and there is growing evidence that exposure to it could be genotoxic, reprotoxic, neurotoxic, etc., for mammals, including humans. In the presented review, we attempted to take a closer look at acetamiprid, its general effects, and its impact on reproduction. Based on the obtained information, it is possible to conclude that acetamiprid causes reproductive toxicity in both male and female individuals. However, further research is needed for a better understanding of the neonicotinoids mechanism of action in higher organisms like mammals because they are still widely used in agriculture. It is particularly necessary to conduct more diverse studies regarding non-target species’ exposure to acetamiprid and gather conclusive evidence. These studies may be helpful in forming an effective strategy and improving the management of acetamiprid applications in the future. In the meantime, environmentally friendly solutions, such as green pesticides and integrated pest control, should be assessed and implemented to see how successful they are against targeted pests. A sustainable strategy to pest control could involve identifying potential acetamiprid treatment sites as well as combining green pesticides and biological controls with a suitable proportion of synthetic pesticides.

## Figures and Tables

**Table 1:** Acute median lethal concentrations (LC50) or lethal doses (LD50) of neonicotinoid exposure for non-target organisms.

Taxon	Research Object	LC50 or LD50	References
Aquatic vertebrates	Fish	1.2–241 mg/L (IMI) >93.6 mg/L (CLO)	Gibbons et al. [67]
Birds	Mallards Grey partridges	283 mg/kg (IMI) 98 mg/kg (ACE) 576 mg/kg (TMX) >752 mg/kg (CLO) 15–41 mg/kg (IMI) 430 mg/kg (CLO)	Mineau and Palmer [68]
Mammals	Rats	Oral 450 mg/kg (IMI) 182 mg/kg (ACE) Oral 1563 mg/kg (TMX) >5000 mg/kg (CLO) Oral 640 mg/kg (THC) 2400 mg/kg (DIN)	Sheets et al. [69]

IMI, Imidacloprid; ACE, acetamiprid; TMX, thiamethoxan; CLO, clothianidin; THC, thiacloprid; DIN, dinotefuran.

**Table 2:** Neurological effects of various neonicotinoids.

Type of Neonicotinoid	Dose	Effect	References
Imidacloprid	45 and 90 mg/kg b.w.	Pain threshold and locomotor activity decreased in rats.	Lonare et al. [70]
	1–100 µM	Cytotoxic to cerebellar neurons: significant excitatory Ca^2+^ influxes were evoked in neonatal rats.	Kimura-Kuroda et al. [58]
Thiamethoxam	50 or 100 mg/kg	Increased anxiety behaviour. HACU and acetylcholinesterase significantly decreased in rats.	Rodrigues et al. [71]
Clothianidin	24 mg/kg	Significant deterioration of cognitive function in infant rats.	Özdemir et al. [72]

**Table 3:** Other toxicity impacts of various neonicotinoids.

Neonicotinoid	Doses	Effect	References
Imidacloprid	>5 mg/kg	Immunosuppressive effect in mice.	Badgujar et al. [75]
	20 mg/kg	Production of radicals and damage to the antioxidant defence system in female rats.	Kapoor et al. [76]
	20 mg/kg/day	Pathomorphological alterations in atretic and antral follicles; decrease in ovarian weight; significant impact to hormone release in female rats.	Kapoor et al. [77]
	1 mg/kg b.w./day	Induced oxidative stress and inflammation in liver and brain of rats.	Duzguner and Erdogan [78]
	8 mg/kg b.w.	Decreased sperm motility and sperm morphology and increased gečrm cell apoptosis in male rats.	Bal et al. [79]
Clothianidin	4 mg/kg b.w./20 mg/kg b.w.	Changes in kidney biochemical parameters in infant and adult male rats.	Ayse Dilek Ozsahin and Okkes [80]

**Table 4:** In vitro studies of acetamiprid exposure resulting in ROS generation.

	Cell Type	Dose	Results	References
In vitro	Pheochromocytoma adrenal medulla cells (PC12)	100–700 µM	-↑ Malondialdehyde levels and ROS generation; loss of mitochondrial membrane potential	Annabi et al. [111]
	Pancreatic cell line (AR42J)	1–6 mM	-Reduction in glutathione levels	Kara et al. [112]
	Isolated trophoblast cells (HTR-8/SVneo)	10 and 100 µM	-↑ ROS production and superoxides; ↓ Glutathione S-transferase, catalase, and superoxide dismutase	Gomez et al. [113]

## Data Availability

Not applicable.
